# 多种肿瘤标志物在肺腺癌中的表达及预后意义的研究

**DOI:** 10.3779/j.issn.1009-3419.2014.03.11

**Published:** 2014-03-20

**Authors:** 欣 杨, 丽燕 薛, 蕾 郭, 芃 温, 冬梅 林

**Affiliations:** 100021 北京，北京协和医学院中国医学科学院肿瘤医院病理科 Department of Pathology, Cancer Institute (Hospital), Chinese Academy of Medical Sciences, Peking Union Medical College, Beijing 100021, China

**Keywords:** 组织芯片, 免疫组化, 肺肿瘤, 预后, Tissue microarray, Immunohistochemistry, Lung neoplasms, Prognosis

## Abstract

**背景与目的:**

肺腺癌是最常见的肺癌组织学类型，发病率呈上升趋势。本研究旨在探讨Napsin A、TTF-1、ERCC1、RRM1、EGFR、HER2、ERα、ERβ、PR、Bcl-2蛋白在肺腺癌中的表达及与临床病理特征和预后的关系。

**方法:**

将227例肺腺癌石蜡标本构建组织芯片。采用免疫组化方法检测10种靶蛋白标记物在肺腺癌中的表达，分析各蛋白表达与患者临床病理特征和预后的关系。

**结果:**

10种蛋白中仅Napsin A蛋白表达与性别有关（*P*=0.049）；Napsin A、PR和EGFR蛋白表达与吸烟有关，TTF-1和ERCC1蛋白表达与肿瘤大小有关，Napsin A、TTF-1、ERα和PR蛋白表达与肿瘤分化程度有关，TTF-1、Bcl-2和ERCC1蛋白表达与病理分期有关（*P* < 0.05）。10种蛋白表达均与年龄无关，ERβ、HER2和RRM1蛋白表达与各项临床病理参数均无相关性（*P* > 0.05）。单因素生存分析显示，Napsin A、TTF-1和ERCC1蛋白表达与患者总生存相关；TTF-1蛋白表达与患者无病生存相关（*P* < 0.05）。进一步分析Ⅰ期肺腺癌，仅Napsin A蛋白表达与患者总生存相关，*P* < 0.05；10种蛋白表达均与Ⅰ期患者无病生存无关（*P* > 0.05）。经多因素生存分析，仅病理分期与患者总生存和无病生存相关（*P* < 0.05）；10种蛋白表达均未显示与患者生存的相关（*P* > 0.05）。

**结论:**

Napsin A、TTF-1和ERCC1蛋白可能是提示肺腺癌患者预后较好的标记物。

## Abstract (en)

**Background and objective:**

Lung adenocarcinoma is one of the most common histological subtypes of lung cancer. The incidence of this disease was continuously increased. This study aims to detect the expressions of Napsin A, TTF-1, ERCC1, RRM1, EGFR, HER2, ERα, ERβ, PR, and Bcl-2 in lung adenocarcinoma and to explore their correlations with clinicopathological characteristics and prognosis.

**Methods:**

A total of 227 lung adenocarcinoma specimens were constructed in tissue microarrays. The expressions of the 10 tumor biomarkers were analyzed by immunohistochemistry on paraffin-embedded sections.

**Results:**

Among the 10 markers, Napsin A was gender-related (*P*=0.049). Napsin A, PR, and EGFR were significantly associated with smoking. TTF-1 and ERCC1 were closely associated with tumor size. Napsin A, TTF-1, ERα, and PR were remarkably associated with tumor differentiation. TTF-1, Bcl-2, and ERCC1 were closely associated with tumor stage (*P* < 0.05). No marker was related to age. No correlations were observed between ERβ, HER2, and RRM1 expressions and clinicopathological parameters (*P* > 0.05). Univariate analysis results showed that Napsin A, TTF-1, and ERCC1 were significantly associated with overall survival. TTF-1 was remarkably associated with disease-free survival (*P* < 0.05). Stage Ⅰ cases were further analyzed and revealed that only Napsin A expression was associated with overall survival (*P* < 0.05). No marker was correlated with disease-free survival (*P* > 0.05). Multivariate analysis results showed that pathological staging was significantly associated with overall survival and disease-free survival (*P* < 0.05). No marker was identified as a predictor of patient outcome (*P* > 0.05).

**Conclusion:**

Napsin A, TTF-1, and ERCC1 are the markers indicating good prognosis of lung adenocarcinoma.

腺癌作为最常见的肺癌组织学类型，在全球及我国发病率均呈上升趋势^[1]^。流行病学研究结果显示，非小细胞肺癌（non-small cell lung cancer, NSCLC）的发病有明显的性别差异，尤其是腺癌。腺癌占女性原发肺癌的75%，是年轻非吸烟患者的主要组织学类型^[2, 3]^，近年与腺癌表达及预后相关标记物的研究已成为热点。本研究中采用免疫组化方法，检测一组标记物在肺腺癌组织中表达情况，分析其表达与临床病理特征和预后的关系。

## 1 材料与方法

### 1.1 临床资料

选择2003年1月-2006年12月中国医学科学院肿瘤医院手术切除（肿瘤及其所在肺叶+区域淋巴结清扫）且随访资料完整，明确诊断肺腺癌的患者227例。患者术前均未行放、化疗。肺癌肿瘤组织经常规甲醛固定、石蜡包埋。按照第7版美国癌症联合委员会（American Joint Committee on Cancer, AJCC）肿瘤分期标准进行TNM分期。记录患者性别、年龄、吸烟史、分期、分化程度、术后治疗（包括化疗、放疗及靶向治疗）及随访情况等。将吸烟史按照吸烟（包括当前吸烟——正吸烟或戒烟＜12个月和既往吸烟——一生累积吸烟量＞100支，肺癌确诊前戒烟＞12个月）和非吸烟（从未吸烟或一生累积吸烟量＜100支）进行分组分析。

### 1.2 材料

组织芯片仪采用Tissue Microarrayer，美国Beecher Instruments公司产品。一抗来源：小鼠单克隆抗体Napsin，Abcam公司产品，原液1:100稀释；小鼠单克隆抗体TTF-1、兔单克隆抗体ERα、兔多克隆抗体ERβ、小鼠单克隆抗体PR、小鼠单克隆抗体Bcl-2及兔多克隆抗体RRM1，北京中杉生物技术公司，工作液；小鼠单克隆抗体HER2及小鼠单克隆抗EGFR野生型抗体，Dako公司，工作液；小鼠单克隆抗体ERCC1，福州迈新生物技术公司产品，工作液。二抗来源：Ventana即用型抗体。

### 1.3 方法

#### 1.3.1 组织芯片（tissue microarray, TMA）的构建

选取并标记HE切片上具有代表性的肿瘤区域及相应蜡块。将所需的靶组织（每例选取2个肿瘤组织芯）用直径1.0 mm的吸针从供体蜡块靶区取出，按芯片设计的排列顺序定位装载在受体蜡块中。受体蜡块连续4 μm切片备用。

#### 1.3.2 免疫组化染色

白片经70 ℃烤箱烤至少1 h，二甲苯脱蜡，梯度酒精水化至蒸馏水后行抗原修复，0.01 mol/L枸橼酸盐缓冲液（pH=6.0，Napsin A、ERα、ERβ、PR、HER2、EGFR、ERCC1）或1 mM EDTA（pH=9.0，TTF-1、RRM1）高压抗原修复2.0 min。一抗室温孵育1 h，通用型二抗室温孵育15 min，二氨基联苯胺显色，苏木素复染。

#### 1.3.3 免疫组化染色结果判定

（1）阳性染色定位：TTF-1、ERα、ERβ、PR、ERCC1阳性染色定位于细胞核，HER2为细胞膜，EGFR为细胞膜和（或）细胞浆，Bcl-2、RRM1为细胞浆。（2）评分标准：①Napsin A、TTF-1、EGFR、Bcl-2、ERCC1、RRM1：（-）无肿瘤细胞着色，（1+）为浅黄色，（2+）为棕黄色，（3+）为黑棕色。③ERα、ERβ、PR：（-）无肿瘤细胞着色或着色肿瘤细胞数＜5%，（1+）着色细胞数为5%-25%，（2+）着色细胞数26%-50%，（3+）着色细胞数＞50%。④HER2：（-）无着色，（1+）任何比例的癌细胞呈现微弱、不完整的细胞膜着色，（2+）＞10%的癌细胞呈现弱至中等强度、完整但不均匀的细胞膜棕黄着色或＜30%的浸润癌细胞呈现强且完整的细胞膜棕褐着色，（3+）＞30%的癌细胞呈现强的、完整的细胞膜棕褐着色。同一例两个组织芯片染色不一致时以高评分为准。由2名病理医师分别阅片，结果一致者为最后判定结果。HER2蛋白染色结果以2+及以上定义为阳性组（+）进行结果分析，其他9种蛋白以1+及以上定义为阳性组（+）进行结果分析。

### 1.4 统计学方法

采用SPSS 20.0统计软件进行统计分析。采用*χ*^2^检验比较不同临床病理特征病例的癌组织中各蛋白表达差异，采用*Kaplan*-*Meier*方法进行单因素生存分析，*Cox*回归进行多因素生存分析。以*P*＜0.05为差异有统计学意义。

## 2 结果

### 2.1 基于组织芯片技术的免疫组化检测有效率

组织芯片制作过程中常出现脱靶（所取靶区肿瘤组织连续切片后消失）及免疫组化染色操作过程中组织样品脱片现象，本研究中10种标记物检测有效率为91.2%-95.6%（表 1）。

**1:** 10种蛋白在组织芯片中的检测有效率 Testing efficiency of 10 markers on TMA sections

Immune markers	Efficiency	Effective number of cases
Napsin A	95.6%	217
TTF-1	93.4%	212
ER*α*	93.0%	211
ER*β*	94.7%	215
PR	93.0%	211
HER2	92.5%	210
EGFR	93.0%	211
Bcl-2	92.5%	210
ERCC1	92.5%	210
RRM1	91.2%	207

### 2.2 临床病理特征和预后

男性137例，女性90例，男女比例为1.52:1。中位年龄61岁。223例患者有吸烟史资料，吸烟者96例，非吸烟者127例。病理分期为Ⅰ期145例，Ⅱ期33例，Ⅲ期45例，Ⅳ期4例。分化程度为高分化39例，中分化109例，低分化79例。术后行辅助治疗34例（Ⅰ期8例：单化疗5例，放、化疗1例，靶向治疗1例，治疗方式不详1例；Ⅱ期、Ⅲ期、Ⅳ期共26例：单放疗3例，单化疗13例，放、化疗10例），5年总生存率和5年无病生存率分别为72.8%和65.3%；未行辅助治疗193例，5年总生存率和5年无病生存率分别为70.2%和61.8%，术后治疗和非治疗组之间总生存和无病生存均无明显差异（*P*＞0.05）。随访时间为3个月-99个月，所有患者的5年总生存率为72.2%，其中Ⅰ期、Ⅱ期、Ⅲ期和Ⅳ期患者的5年总生存率分别为88.1%、63.1%、33.3%和0，差异有统计学意义（*P*＞0.001）。

### 2.3 蛋白在肺腺癌中的表达与患者临床病理特征的关系

免疫组化方法检测10种蛋白在肺腺癌组织中的表达情况见图 1。其中仅Napsin A蛋白表达与性别有关（*P=*0.049）。Napsin A、PR及EGFR蛋白表达与吸烟有关，TTF-1和ERCC1蛋白表达与肿瘤大小有关，Napsin A、TTF-1、ERα和PR蛋白表达与分化程度有关，表达与病理分期有关的是TTF-1、Bcl-2和ERCC1蛋白（*P*＜0.05）。未观察到上述蛋白表达与患者年龄有关，ERβ、HER2和RRM1蛋白表达与性别、年龄、吸烟、肿瘤大小、分化程度及病理分期均无关性（*P*＞0.05）（表 2）。

**1:**
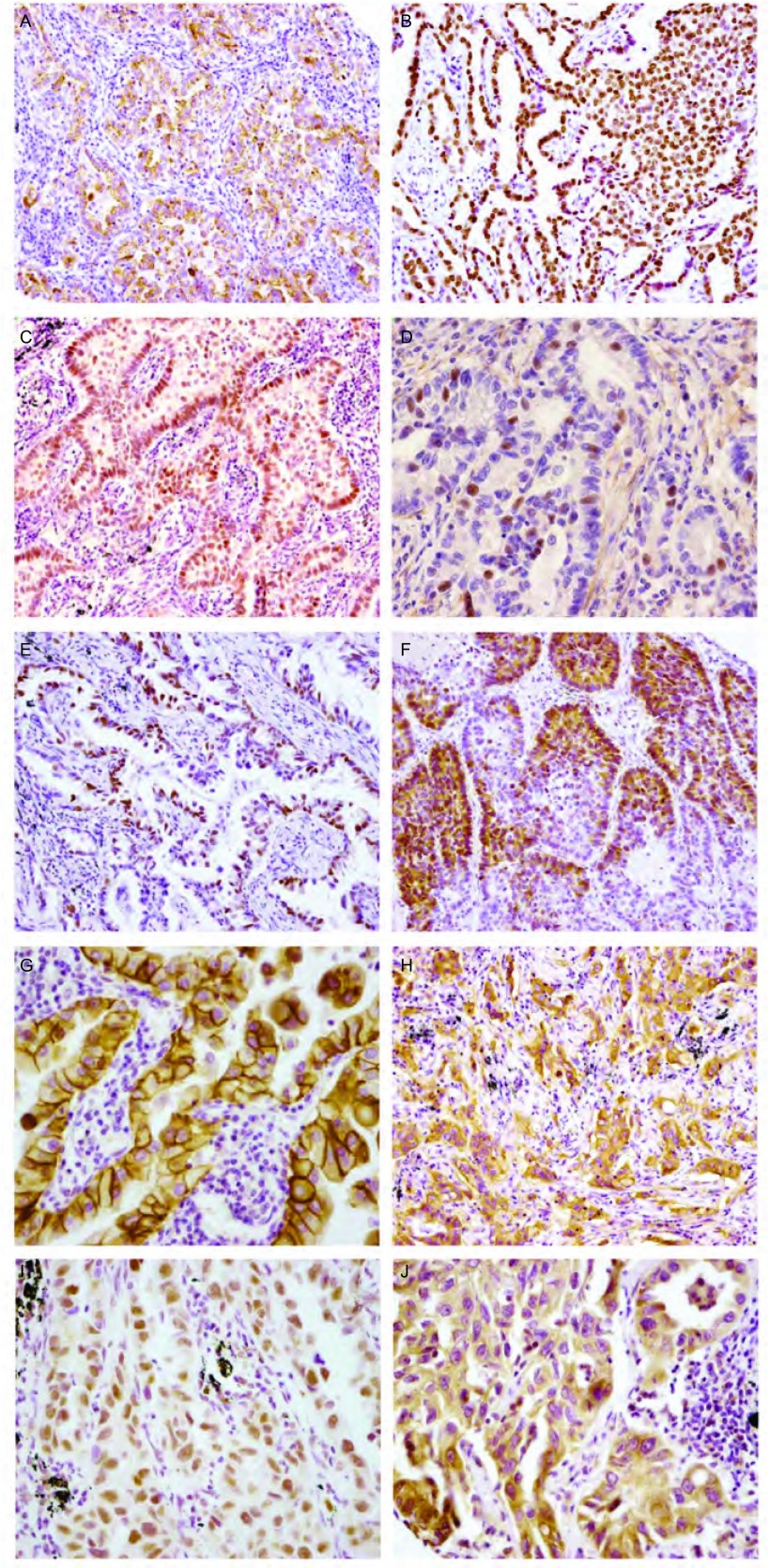
免疫组化方法检测10种蛋白在肺腺癌组织中的表达。A：Napsin A（×200）；B：TTF1（×200）；C：ER*α*（×200）；D：ER*β*（×400）；E：PR（×200）；F：Bcl-2（×200）；G：HER2（×400）；H：EGFR（×200）；I：ERCC1（×400）；J：RRM1（×400）。 Expression of 10 markers in lung adenocarcinoma analyzed by immunohistochemistry. A: Napsin A (×200); B: TTF1 (×200); C: ER*α* (×200); D: ER*β* (×400); E: PR (×200); F: Bcl-2 (×200); G: HER2 (×400); H: EGFR (×200); I: ERCC1 (×400); J: RRM1 (×400).

**2:**
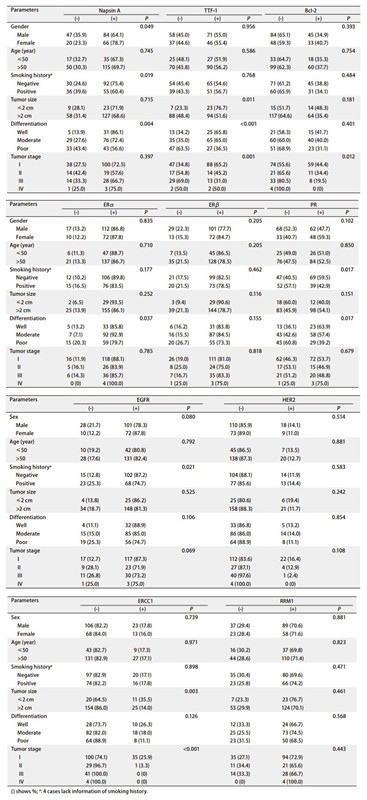
10种蛋白表达及与患者临床病理特征的关系 The relationship between expression of 10 tumor biomarkers and clinicopathological characteristics in the patients with lung cancer

Parameters	Napsin A				TTF-1				Bcl-2				ER*α*				ER*β*				PR				EGFR				HER2				EGFR				HER2		
	(-)	(+)	*P*		(-)	(+)	*P*		(-)	(+)	*P*		(-)	(+)	*P*		(-)	(+)	*P*		(-)	(+)	*P*		(-)	(+)	*P*		(-)	(+)	*P*		(-)	(+)	*P*		(-)	(+)	*P*
Gender			0.049				0.956				0.393				0.835				0.205				0.102				0.080				0.514				0.739				0.881
Male	47 (35.9)	84 (64.1)			58 (45.0)	71 (55.0)			84 (65.1)	45 (34.9)			17 (13.2)	112 (86.8)			29 (22.3)	101 (77.7)			68 (52.3)	62 (47.7)			28 (21.7)	101 (78.3)			110 (85.9)	18 (14.1)			106 (82.2)	23 (17.8)			37 (29.4)	89 (70.6)	
Female	20 (23.3)	66 (78.7)			37 (44.6)	46 (55.4)			48 (59.3)	33 (40.7)			10 (12.2)	72 (87.8)			13 (15.3)	72 (84.7)			33 (40.7)	48 (59.3)			10 (12.2)	72 (87.8)			73 (89.0)	9 (11.0)			68 (84.0)	13 (16.0)			23 (28.4)	58 (71.6)	
Age (year)			0.745				0.586				0.754				0.710				0.205				0.850				0.792				0.881				0.971				0.823
≤50	17 (32.7)	35 (67.3)			25 (48.1)	27 (51.9)			33 (64.7)	18 (35.3)			6 (11.3)	47 (88.7)			7 (13.5)	45 (86.5)			25 (49.0)	26 (51.0)			10 (19.2)	42 (80.8)			45 (86.5)	7 (13.5)			43 (82.7)	9 (17.3)			16 (30.2)	37 (69.8)	
> 50	50 (30.3)	115 (69.7)			70 (43.8)	90 (56.2)			99 (62.3)	60 (37.7)			21 (13.3)	137 (86.7)			35 (21.5)	128 (78.5)			76 (47.5)	84 (52.5)			28 (17.6)	131 (82.4)			138 (87.3)	20 (12.7)			131 (82.9)	27 (17.1)			44 (28.6)	110 (71.4)	
Smoking history^a^			0.019				0.768				0.484				0.177				0.462				0.017				0.021				0.583				0.898				0.471
Negative	30 (24.6)	92 (75.4)			54 (45.4)	65 (54.6)			71 (61.2)	45 (38.8)			12 (10.2)	106 (89.8)			21 (17.5)	99 (82.5)			47 (40.5)	69 (59.5)			15 (12.8)	102 (87.2)			104 (88.1)	14 (11.9)			97 (82.9)	20 (17.1)			35 (30.4)	80 (69.6)	
Positive	36 (39.6)	55 (60.4)			39 (43.3)	51 (56.7)			60 (65.9)	31 (34.1)			15 (16.5)	76 (83.5)			20 (21.5)	73 (78.5)			52 (57.1)	39 (42.9)			23 (25.3)	68 (74.7)			77 (85.6)	13 (14.4)			74 (82.2)	16 (17.8)			23 (25.8)	66 (74.2)	
Tumor size			0.715				0.011				0.181				0.252				0.116				0.151				0.525				0.242				0.003				0.461
≤2 cm	9 (28.1)	23 (71.9)			7 (23.3)	23 (76.7)			15 (51.7)	14 (48.3)			2 (6.5)	29 (93.5)			3 (9.4)	29 (90.6)			18 (60.0)	12 (40.0)			4 (13.8)	25 (86.2)			25 (80.6)	6 (19.4)			20 (64.5)	11 (35.5)			7 (23.3)	23 (76.7)	
> 2 cm	58 (31.4)	127 (68.6)			88 (48.4)	94 (51.6)			117 (64.6)	64 (35.4)			25 (13.9)	155 (86.1)			39 (21.3)	144 (78.7)			83 (45.9)	98 (54.1)			34 (18.7)	148 (81.3)			158 (88.3)	21 (11.7)			154 (86.0)	25 (14.0)			53 (29.9)	124 (70.1)	
Differentiation			0.004				< 0.001				0.401				0.037				0.155				0.017				0.106				0.854				0.126				0.568
Well	5 (13.9)	31 (86.1)			13 (34.2)	25 (65.8)			21 (58.3)	15 (41.7)			5 (13.2)	33 (85.8)			6 (16.2)	31 (83.8)			13 (36.1)	23 (63.9)			4 (11.1)	32 (88.9)			33 (86.8)	5 (13.2)			28 (73.7)	10 (26.3)			12 (33.3)	24 (66.7)	
Moderate	29 (27.6)	76 (72.4)			35 (35.0)	65 (65.0)			60 (60.0)	40 (40.0)			7 (7.1)	92 (92.9)			16 (15.5)	87 (84.5)			43 (42.6)	58 (57.4)			15 (15.0)	85 (85.0)			86 (86.0)	14 (14.0)			82 (82.0)	18 (18.0)			25 (25.5)	73 (74.5)	
Poor	33 (43.4)	43 (56.6)			47 (63.5)	27 (36.5)			51 (68.9)	23 (31.1)			15 (20.3)	59 (79.7)			20 (26.7)	55 (73.3)			45 (60.8)	29 (39.2)			19 (25.3)	56 (74.7)			64 (88.9)	8 (11.1)			64 (88.9)	8 (11.1)			23 (31.5)	50 (68.5)	
Tumor stage			0.397				0.001				0.012				0.785				0.818				0.679				0.069				0.108				< 0.001				0.443
Ⅰ	38 (27.5)	100 (72.5)			47 (34.8)	88 (65.2)			74 (55.6)	59 (44.4)			16 (11.9)	118 (88.1)			26 (19.0)	111 (81.0)			62 (46.3)	72 (53.7)			17 (12.7)	117 (87.3)			112 (83.6)	22 (16.4)			100 (74.1)	35 (25.9)			35 (27.1)	94 (72.9)	
Ⅱ	14 (42.4)	19 (57.6)			17 (54.8)	14 (45.2)			21 (65.6)	11 (34.4)			5 (16.1)	26 (83.9)			8 (25.0)	24 (75.0)			17 (53.1)	15 (46.9)			9 (28.1)	23 (71.9)			27 (87.1)	4 (12.9)			29 (96.7)	1 (3.3)			11 (34.4)	21 (65.6)	
Ⅲ	14 (33.3)	28 (66.7)			29 (69.0)	13 (31.0)			33 (80.5)	8 (19.5)			6 (14.3)	36 (85.7)			7 (16.7)	35 (83.3)			21 (51.2)	20 (48.8)			11 (26.8)	30 (73.2)			40 (97.6)	1 (2.4)			41 (100.0)	0 (0)			14 (33.3)	28 (66.7)	
Ⅳ	1 (25.0)	3 (75.0)			2 (50.0)	2 (50.0)			4 (100.0)	0 (0)			0 (0)	4 (100.0)			1 (25.0)	3 (75.0)			1 (25.0)	3 (75.0)			1 (25.0)	3 (75.0)			4 (100.0)	0 (0)			4 (100.0)	0 (0)			0 (0)	4 (100.0)	
() shows %; ^a^: 4 cases lack information of smoking history. Table 2 continues																																							

### 2.4 生存分析

单因素生存分析评估临床病理特征与预后关系，其中肿瘤大小、分化程度和病理分期与总生存（overall survival, OS）和无病生存（disease-free survival, DFS）有关（*P*＜0.05）（表 3）；单因素生存分析10种蛋白在肿瘤组织中表达与预后：Napsin A、TTF-1和ERCC1蛋白表达与OS有关（图 2A-图 2C）；TTF-1蛋白表达与患者DFS有关（图 2D）（*P*＜0.05）（表 4）。进一步对Ⅰ期患者进行分析，仅Napsin A蛋白表达与Ⅰ期患者OS有关（图 2E）（*P*＜0.05）。未观察到表达与Ⅰ期患者无病生存有关的蛋白（*P*＞0.05）（表 5）。多因素生存分析，仅病理分期是患者总生存和无病生存相关的预后因素（*P*＜0.05），未发现表达与患者预后相关的蛋白（*P*＞0.05）（表 6）。

**3:** 单因素分析临床病理特征与5年总生存率和5年无病生存率关系 The relationship among the clinicopathologic characteristics, 5-year overall survival (OS) and 5-year disease-free survival (DFS) analyzed by univariate analysis

Parameters	*n*	5-year OS (%)	*P*	5-year DFS (%)	*P*
Sex			0.859		0.292
Male	137	72.3		63.1	
Female	90	72.4		67.1	
Age (year)			0.365		0.556
≤50	57	77.2		69.2	
> 50	170	70.4		62.9	
Smoking history^a^			0.202		0.431
Negative	127	76.2		67.2	
Positive	96	69.1		62.9	
Tumor size			0.001		0.001
≤2 cm	33	97.0		90.6	
> 2 cm	194	67.9		60.2	
Differentiation			< 0.001		0.009
Well	39	89.7		79.8	
Moderate	109	77.1		68.3	
Poor	79	55.7		51.4	
Tumor stage			< 0.001		< 0.001
Ⅰ	145	88.1		78.2	
Ⅱ	33	63.1		53.3	
Ⅲ	45	33.3		16.2	
Ⅳ	4	0		0	

**2:**
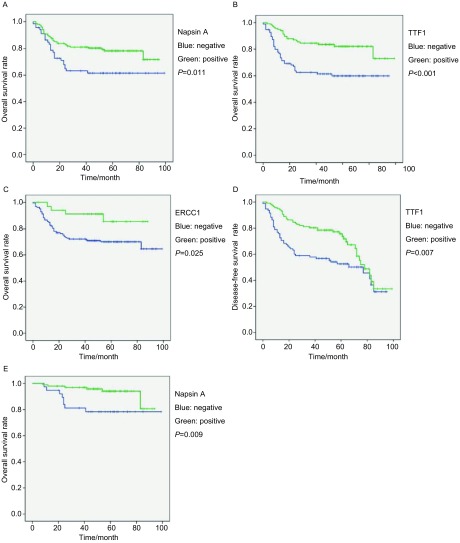
生存曲线。A：全部病例Napsin A蛋白总生存曲线；B：全部病例TTF1蛋白总生存曲线；C：全部病例ERCC1蛋白总生存曲线；D：全部病例TTF1蛋白无病生存曲线；E：Ⅰ期病例Napsin A蛋白总生存曲线。 Survival curves. A: OS curves for Napsin A in all cases; B: OS curves for TTF1 in all cases. C: OS curves for ERCC1 in all cases; D: DFS curves for TTF1 in all cases; E: OS curves for Napsin A in stage Ⅰ cases.

**4:** 单因素分析各蛋白表达与5年总生存率和5年无病生存率关系 The relationship among the expression of tumor markers, 5-year overall survival (OS) and 5-year disease-free survival (DFS) analyzed by univariate analysis

Variables	*n*	5-year OS (%)	*P*	5-year DFS (%)	*P*
Napsin A			0.011		0.127
(-)	67	61.6		56.3	
(+)	150	78.3		70.4	
TTF-1			＜0.001		0.007
(-)	95	59.8		45.9	
(+)	117	82.1		68.6	
ER*α*			0.263		0.261
(-)	27	65.4		59.3	
(+)	184	73.6		66.5	
ER*β*			0.663		0.676
(-)	42	70.4		58.8	
(+)	173	73.7		67.5	
PR			0.714		0.973
(-)	101	72.6		61.5	
(+)	110	71.3		67.6	
Bcl-2			0.138		0.115
(-)	132	69.4		62.5	
(+)	78	77.8		70.4	
EGFR			0.693		0.930
(-)	38	70.6		64.6	
(+)	173	73.5		66.3	
HER2			0.849		0.300
(-)	183	72.8		65.2	
(+)	27	73.3		70.4	
ERCC1			0.025		0.091
(-)	174	70.0		63.2	
(+)	36	85.6		78.0	
RRM1			0.252		0.149
(-)	60	66.7		59.1	
(+)	147	75.1		68.4	

**5:** 单因素分析各蛋白表达与Ⅰ期患者5年总生存率和5年无病生存率 The relationship among the expression of tumor markers, 5-year overall survival (OS) and 5-year disease-free survival (DFS) in patients with stage Ⅰ disease analyzed by univariate analysis

Variables	*n*	5-year OS (%)	*P*	5-year DFS (%)	*P*
Napsin A			0.009		0.219
(-)	38	78.3		73.0	
(+)	100	94.1		83.5	
TTF-1			0.089		0.182
(-)	47	82.3		70.6	
(+)	88	92.2		79.9	
ER*α*			0.629		0.806
(-)	16	87.1		81.3	
(+)	118	89.8		80.8	
ER*β*			0.785		0.997
(-)	26	88.1		76.0	
(+)	111	90.1		81.6	
PR			0.746		0.644
(-)	62	89.7		74.8	
(+)	72	88.3		83.7	
Bcl-2			0.710		0.329
(-)	74	88.8		78.7	
(+)	59	90.5		83.2	
EGFR			0.232		0.397
(-)	17	81.4		71.3	
(+)	117	90.7		82.0	
HER2			0.154		0.915
(-)	112	91.1		80.6	
(+)	22	81.8		81.8	
ERCC1			0.876		0.992
(-)	100	90.6		81.1	
(+)	35	85.6		80.3	
RRM1			0.121		0.116
(-)	35	83.6		71.5	
(+)	94	92.3		84.6	

**6:** 多因素分析病理特征和蛋白表达与肺腺癌患者预后关系 The correlations among clinicopathologic features and tumor marker expression with 5-year overall survival (OS)/5-year disease-free survival (DFS) in the patients with adenocarcinoma analyzed by mutivariate analysis

Variables	5-year OS RR (95%CI)	*P*	5-year DFS RR (95%CI)	*P*
Tumor size	281028 (0.000-∞)	0.963	2.138 (0.758-6.030)	0.151
Differentiation	1.050(0.475-2.319)	0.905	1.288 (0.915-1.813)	0.147
Tumor stage	2.674 (1.965-3.641)	< 0.001	1.886 (1.491-2.386)	< 0.001
Naspin A	0.713 (0.327-1.553)	0.394	-	-
ERCC1	3.697 (1.142-11.964)	0.895	-	-
TTF-1	-	-	0.862 (0.550-1.350)	0.516
- is variables not included in the analysis.				

## 3 讨论

目前TNM分期是评估肺癌预后最重要的指标，但相同分期患者预后存在显著差别，即使根治性切除的Ⅰ期肺腺癌病例，5年复发率也有40%^[4]^。因此，寻找可以提示预后的分子病理标志物是肺癌研究亟待解决的问题。

TTF-1和Napsin A作为肺腺癌敏感性及特异性均较好的标记物，临床最常组合应用于肺腺癌组织学亚型的鉴别诊断中，近年TTF-1还被视作一个潜在的NSCLC预后标记物，高表达者预后好^[5, 6]^。本研究结果显示TTF-1表达与5年总生存及无病生存均相关，高表达者预后好，与上述文献报道一致。同时还发现TTF-1蛋白表达与肿瘤大小、分化程度和病理分期相关，在最大径≤2 cm、高分化和Ⅰ期患者肿瘤组织中表达率高，而以上3项临床病理特征均是公认提示预后好的因素，也间接为TTF-1高表达提示患者预后好提供佐证。Napsin A是肺和肾组织中均有表达的天冬氨酸蛋白酶，能够分离表达于Ⅱ型肺泡上皮的表面活性蛋白B（SP-B）前体。随着肺腺癌分子检测和靶向治疗的进展，使NSCLC亚型分类的意义愈发明显，目前Napsin A在NSCLC鉴别腺癌亚型中的应用较广泛，然而至今关于Napsin A表达与肺腺癌预后的意义研究较少。本研究发现Napsin A蛋白表达与性别、吸烟史及肿瘤分化程度有关，女性、非吸烟者及高分化者阳性率高，且不论在所有病期还是Ⅰ期病例中阳性患者总生存率高于阴性者，结果与Lee等^[7]^研究一致。

ERα、ERβ及PR是类固醇受体，属于核受体超家族。雌、孕激素受体不仅存在于乳腺、子宫等雌激素靶器官，近年来的研究^[8]^表明，在NSCLC和正常肺组织中亦有表达，且表达与特定的临床病理特征与预后有关。本研究显示ERα及PR蛋白表达均与肿瘤分化程度有关，分化好者高表达，PR蛋白表达还与吸烟有关，非吸烟者阳性率高，但二者表达均未显示与预后有关。ERβ蛋白表达既与临床病理特征无关也未发现有预后提示意义。包括本研究在内，NSCLC或肺腺癌中ERα、ERβ和PR蛋白表达与临床病理特征及预后关系的相关研究结果尚存争议^[9-12]^，可能与实验设计的选择偏倚、实验条件差异及抗体克隆选择不同有关，也可能与不同研究组免疫组化结果判读标准差异有关。尚需更多研究探索它们在肺腺癌发生和发展中的意义。

*Bcl*-*2*是一种癌基因，其蛋白产物是细胞凋亡蛋白家族成员之一，分子量25 kDa的线粒体内膜蛋白，表达于一系列淋巴组织增生性疾病和包括肺癌在内的多种非造血系统恶性肿瘤中。Bcl-2蛋白抑制细胞凋亡，因此认为该蛋白过表达的肿瘤可能会获得生长优势。Bcl-2蛋白表达与肺癌临床病理特征及预后关系研究不多，本研究中Bcl-2蛋白表达与肿瘤分期有关，病期晚者阳性率高，但生存分析未显示其表达对患者预后有提示意义，结果与Yaren等^[13]^和Hanaoka等^[14]^报道一致。

EGFR和HER-2/neu均为表皮生长因子受体家族成员，在细胞信号传到中发挥重要的作用，是细胞生长、分化及凋亡的重要调节者。该家族包括EGFR（HER1或erbB1）、HER2（neu或erbB2）、HER3（erbB3）及HER4（tyro2或erbB4）。近年肺腺癌治疗和预后相关研究最重要的分子事件是EGFR突变，主要集中在外显子19（A746-A750）缺失突变和外显子21（L858R）点突变，是晚期肺腺癌一线EGFR-酪氨酸激酶抑制剂治疗疗效的预测因子^[15]^。还有研究^[16]^报道即使接受传统的化疗，*EGFR*突变阳性的患者也显示较好的临床转归，因此提示*EGFR*突变为提示预后好的分子指标。而野生型EGFR蛋白反映*EGFR*基因扩增状态，与*EGFR*突变情况无平行关系，因此*EGFR*基因突变的预后价值不能代表野生型EGFR蛋白表达与肺腺癌预后的关系。本研究EGFR抗体为抗野生型*EGFR*基因蛋白，结果显示其表达与吸烟有关，非吸烟患者阳性率高，但未观察到野生型EGFR蛋白表达与患者预后相关，提示相对*EGFR*基因拷贝数的增加，*EGFR*基因突变对于评估肺腺癌预后可能更有意义。虽然近年来HER-2/neu在乳腺癌中得到广泛研究，且分别针对基因扩增状态及蛋白表达情况的荧光原位杂交（fluorescence *in situ* hybridization, FISH）和免疫组化检测技术临床应用均已成熟，但目前在肺癌中HER2蛋白表达情况及预后价值信息较少。一项HER2与肺癌预后关系的荟萃分析^[17]^中，纳入荟萃分析的多数免疫组化方法研究以染色结果2+及以上为HER2蛋白阳性判定值，结论显示HER2蛋白过表达提示肺癌尤其小细胞肺癌、肺腺癌和早期NSCLC预后不良。本研究以免疫组化染色结果2+及以上定义为HER2阳性组进行统计分析，结果显示肺腺癌中HER2蛋白表达与各临床病理因素均无相关性，阳性组与阴性组生存率亦无统计学差异，结果可能受到研究样本量小、样本来源单中心及对免疫组化方法检测的HER2蛋白阳性定义标准等限制，HER2蛋白在肺腺癌中表达阳性判定标准及其对肺腺癌患者的预后意义需进一步研究和探讨。

ERCC1蛋白是核苷酸切除修复通路中高度保守的单链DNA核酸内切酶，参与识别和切除DNA加合物，在核苷酸切除修复中起到限速或调节的重要作用。RRM1是核糖核苷酸还原酶的调节单位，参与减少DNA合成和修复需要的核苷酸向脱氧核糖核蛋白的转化。RRM1还增加10号染色体磷酸酶和张力蛋白缺失，导致肿瘤细胞生长，迁移和转移潜能降低，是新型化疗吉西他滨的重要靶点。近年来有研究^[18, 19]^报道肺癌组织中ERCC1和RRM1蛋白表达与治疗反应和患者预后的关系等。2009美国国立综合癌症网络（National Comprehensive Cancer Network, NCCN）推荐ERCC1和RRM1蛋白表达与*EGFR*基因突变一起，作为评估肺癌预后与预测治疗的标记物。但最近Friboulet等^[20]^研究却发现，多种市售ERCC1抗体不能有效识别同时具备核苷酸切除修复功能和铂类抵抗的全能型异构体，因此通过免疫组化方法获得的ERCC1蛋白表达情况，在不同研究或相同病例不同次检测中的重复性差，导致其预后和预测作用有限。本研究观察到ERCC1蛋白表达与肿瘤大小和病理分期有关，肿瘤小和早期患者阳性率高，并且表达与预后有关，阳性组5年总生存率明显高于阴性组，提示ERCC1是提示肺腺癌预后好的标记物，但未多次或选择不同病例库验证。本研究中RRM1蛋白表达与临床病理特征及预后均无关，推测可能亦存在抗体与蛋白异构体识别特异性差的问题，另外在免疫组化染色结果判读中还发现，RRM1抗体的非特异性着色问题较其他抗体明显，尚需优化技术平台进一步论证其与临床预后的关系。

综上，免疫组化方法检测相关标记物表达以评估肺腺癌预后有一定临床应用价值：TTF-1、Napsin A和ERCC1蛋白可能是提示肺腺癌患者预后较好的标记物。
